# DNAJC17 deficiency: A novel inborn error of immunity with TNF-driven autoinflammation

**DOI:** 10.3389/fimmu.2026.1751990

**Published:** 2026-07-09

**Authors:** Fayhan Alroqi, Abdullah Almojali, Abdulrahman N. AlJaber, Nouf Althubaiti, Shouq Alzaaqi, Asayel Almohammadi, Sarah Albahkali, Mahmoud A. Majeed, Abeer Al Tuwaijri, Yasser Basmaeil, Abduarahman Almutairi, Abdulrahman Alrasheed

**Affiliations:** 1 Division of Pediatric Allergy and Immunology, Department of Pediatrics, King Abdullah Specialized Children's Hospital, King Abdulaziz Medical City, Riyadh, Saudi Arabia; 2 King Abdullah International Medical Research Center, Ministry of National Guard–Health Affairs, Riyadh, Saudi Arabia; 3 College of Medicine, King Saud bin Abdulaziz University for Health Sciences, Riyadh, Saudi Arabia; 4 Division of Pediatric Rheumatology, Department of Pediatrics, King Abdullah Specialized Children's Hospital, King Abdulaziz Medical City, Riyadh, Saudi Arabia; 5 Department of Pediatrics, King Fahad Armed Forces Hospital, Jeddah, Saudi Arabia; 6 Clinical Laboratory Sciences Department, College of Applied Medical Sciences, King Saud bin Abdulaziz University for Health Sciences, Ministry of National Guard–Health Affairs, Riyadh, Saudi Arabia

**Keywords:** autoinflammation, DNAJC17 deficiency, inborn errors of immunity (IEI), retinitis pigmentosa, TNF blockade

## Abstract

**Background:**

Inborn Errors of Immunity (IEI) encompass a broad spectrum of monogenic disorders with variable clinical presentations, including recurrent infections, autoimmunity, and systemic inflammation. Loss-of-function mutations in *DNAJC17* have been previously linked to retinal dystrophy and hypogammaglobulinemia; however, the complete immunological phenotype and underlying autoinflammatory mechanisms remain poorly characterized.

**Methods:**

We conducted a comprehensive clinical, molecular, and immunological investigation of three affected individuals from two unrelated consanguineous families. Our evaluation included whole genome sequencing with systematic exclusion of alternative genetic etiologies, quantitative analysis of DNAJC17 mRNA expression, and assessment of intracellular DNAJC17 protein levels. In addition, we performed in-depth immunophenotyping of adaptive and innate immune cell subsets, cytokine profiling, and interferon-stimulated gene expression analysis, alongside longitudinal evaluation of responses to therapeutic interventions.

**Results:**

All three patients presented with early-onset retinitis pigmentosa, recurrent fever, lymphadenitis, and hypogammaglobulinemia. Sanger sequencing confirmed a homozygous variant (c.681G>A; p.Ala227=) in *DNAJC17* resulting in exon 9 skipping. Comprehensive whole genome analysis excluded pathogenic variants in other immune-related genes. Quantitative RT-PCR demonstrated reduced DNAJC17 mRNA levels and flow cytometry showed decreased intracellular protein levels, consistent with a hypomorphic loss-of-function effect. Immunological evaluation revealed distinct abnormalities in T, B, and NK-cell subsets, with altered monocyte and dendritic cell populations indicating innate and adaptive immune dysregulation. Inflammatory profiling identified increased cytokine activity, including TNF-α and IL-6, alongside mild induction of interferon-stimulated genes. Conventional immunomodulatory therapies failed to achieve sustained remission. In contrast, TNF inhibitor therapy resulted in dramatic clinical improvement with normalization of inflammatory markers, sustained for over three years without significant adverse events.

**Conclusion:**

Our findings establish DNAJC17 deficiency as a novel monogenic inborn error of immunity characterized by retinopathy, combined immunodeficiency, and TNF-driven autoinflammation responsive to targeted TNF blockade. The underlying immune abnormalities underscore the essential role of DNAJC17 in maintaining immune homeostasis and mitochondrial function. This work provides mechanistic insights into disease pathogenesis and establishes a rational, precision medicine therapeutic approach for this newly defined syndrome.

## Introduction

Inborn Errors of Immunity (IEI) constitute a heterogeneous group of inherited disorders that predispose affected individuals to recurrent infections, autoimmunity, autoinflammation, allergic manifestations, or malignancies (1). The most recent classification by the International Union of Immunological Societies (IUIS) collectively lists a total of 559 distinct IEIs (2). The clinical manifestations of these disorders are frequently complex, often presenting with overlapping features of classical immunodeficiency, immune dysregulation, and autoinflammation. This phenotypic complexity poses significant diagnostic challenges and blurs traditional distinctions between primary immunodeficiencies and rheumatological conditions (2).

An emerging pathogenic mechanism across various IEIs involves disruption of protein folding and associated cellular stress responses. The DNAJ (Hsp40) family comprises highly conserved co-chaperone proteins that regulate the function of Hsp70 molecular chaperones, directing them to act on specific target proteins or within particular cellular compartments. DNAJC17, a member of the DNAJ subclass C family, is characterized by a distinctive N-terminal J-domain and C-terminal RNA recognition motif (RRM) (3, 4). Studies in murine models have demonstrated that DNAJC17 is highly expressed in the developing thyroid bud and is crucial for proper thyroid organogenesis, with complete knockout resulting in embryonic lethality, highlighting its fundamental importance for cellular viability (4).

Mutations in *DNAJC17* have been associated with several human diseases (5). Two independent studies have reported distinct missense mutations in exon 11 of the human *DNAJC17* gene in patients with essential thrombocythemia (6, 7). In 2016, Patel et al. identified a homozygous synonymous variant (c.681G>A) in two siblings presenting with early-onset retinitis pigmentosa and hypogammaglobulinemia, demonstrating that the variant caused exon 9 skipping through disruption of the splice donor site (8). The same patients were subsequently reported in a later article in the context of COVID-19 infection (9). Additionally, differential alternative splicing of *DNAJC17* has been observed in patients with autism spectrum disorders, though the functional significance remains unclear (10).

Despite these clinical associations, the precise immunological phenotype of DNAJC17 deficiency has not been systematically characterized, and the autoinflammatory component has not been previously recognized or described. The molecular mechanisms linking DNAJC17 deficiency to immune dysregulation remain poorly understood. Here, we present a comprehensive clinical, immunological, and molecular characterization of three patients from two unrelated consanguineous families with DNAJC17 deficiency. Our findings reveal a novel IEI characterized by retinitis pigmentosa, combined immunodeficiency, and TNF-driven autoinflammation, with a remarkable therapeutic response to TNF blockade. This report will expand the clinical and mechanistic understanding of DNAJC17-related diseases and provide critical therapeutic guidance for patient management.

## Materials and methods

### Patients recruitment and clinical evaluation

Three patients from two unrelated consanguineous families were enrolled in this study following informed consent in accordance with protocols approved by the institutional review board (IRB number: RC20/156/R). Detailed clinical histories, physical examinations, and comprehensive laboratory evaluations were performed at regular intervals. Clinical data including growth parameters, infection history, inflammatory episodes, and treatment responses were systematically documented.

### Whole genome sequencing and analysis

Genomic DNA was extracted from peripheral blood using standard protocols. Whole-genome sequencing (WGS) was performed. All variants were classified according to the American College of Medical Genetics and Genomics (ACMG) and the Association for Molecular Pathology (AMP) guidelines as pathogenic, likely pathogenic, variant of uncertain significance, likely benign, or benign. Reporting was restricted to variants in genes with established gene-disease associations (OMIM^®^) and with relevance to the patients’ phenotypes. Copy number variants (CNVs) of uncertain significance were not reported.

### Sanger sequencing of the candidate variant

Variant-specific primers were designed using Primer3 software to amplify the flanking region containing the variant. Sanger sequencing was then carried out using standard methods (11).

### Quantitative RT-PCR for DNAJC17 expression

Total RNA was extracted from whole blood using the RNeasy Mini Kit (Qiagen, Cat. No. 74104, Germany), according to the manufacturer’s instructions. Complementary DNA (cDNA) was synthesized using the High-Capacity cDNA Reverse Transcription Kit (Applied Biosystems™, Thermo Fisher Scientific, Cat. No. 4368813, USA) in a 20 µL reaction volume, following the manufacturer’s protocol. To evaluate the potential splicing effect of the identified *DNAJC17* variant, quantitative real-time RT-PCR (qRT-PCR) was performed. GAPDH was used as the endogenous housekeeping control, and a no-template control was included as a negative control. The region spanning exons 9–10 of *DNAJC17* was amplified using the following primers: forward 5′-TGAGGTTCTCAACCTGGTGC-3′ and reverse 5′-GGTTATCCACCAGGCCAACT-3′, generating an expected amplicon size of 116 bp. All reactions were performed in triplicate using SYBR Green Master Mix on the QuantStudio 6 Flex Real-Time PCR System (Applied Biosystems™, Thermo Fisher Scientific, USA), according to the manufacturer’s instructions. Relative gene expression was calculated using the 2^-^ΔΔCt method, and data were analyzed using GraphPad Prism software version 8.1 (12).

### Flow cytometric analysis for DNAJC17 protein

Indirect intracellular staining for DNAJC17 was performed on isolated peripheral blood mononuclear cells (PBMCs) using BD Biosciences Fixation/Permeabilization buffer with polyclonal rabbit anti-DNAJC17 antibodies (Invitrogen) or Rabbit IgG XP (R) isotype control (cell signaling), followed by secondary detection with Brilliant Violet 421^™^ Donkey anti-Rabbit IgG (Biolegend).

### Interferon-stimulated gene expression analysis

Expression of interferon-stimulated genes (ISGs) was quantified by qPCR using the same cDNA samples. Target genes analyzed were IFI44L, RSAD2, IFI27, and IFIT1, with HPRT1 serving as the endogenous control gene. Gene expression was quantified by qPCR and presented as mean ± standard error of the mean (SEM) of fold change (2^-^ΔΔCt), normalized to the housekeeping gene and relative to the control group. A fold change of ≥ 2 or ≤ 0.5 was considered biologically significant. Statistical analyses were performed on ΔCt values using Welch’s unpaired *t*-test for comparisons between the control and patient 3B off medication, and paired *t*-test for comparisons before and after infliximab treatment. A p-value <0.05 was considered statistically significant.

### Enzyme-linked immunosorbent assay

Serum concentrations of tumor necrosis factor-alpha (TNF-α) and interleukin-6 (IL-6) were quantified using commercially available human ELISA kits (Thermo Fisher Scientific, Waltham, MA, USA) according to the manufacturer’s protocols. The reference ranges for these cytokines are TNF-α < 0.1 pg/mL and IL-6 < 7 pg/mL.

### Immunophenotyping

Comprehensive immunophenotyping was performed on fresh PBMCs. The protocol of the extracellular and intracellular staining was described in our previous report (13). Multicolor flow cytometry was performed using the following antibody panels:

T Cell Panel: Anti-CD3-APC-H7 (clone SK7, BD Biosciences), Anti-CD4-PerCP-Cy5.5 (clone RPA-T4, BD Biosciences), Anti-CD8-V450 (clone RPA-T8, BD Biosciences), Anti-CCR7-PE-Cy7 (clone G043H7, BioLegend), Anti-CD45RA-FITC (clone HI100, BD Biosciences).

B Cell Panel: Anti-CD19-APC-H7 (clone SJ25C1, BD Biosciences), Anti-CD27-PE (clone M-T271, BD Biosciences), Anti-IgD-FITC (clone IA6-2, BD Biosciences).

Natural Killer (NK) Cell Panel: Anti-CD3-FITC (clone SK7, BD Biosciences), Anti-CD56-APC (clone NCAM16.2, BD Biosciences), Anti-CD16-PE (clone B73.1, BD Biosciences).

Monocytes Panel: Anti-CD14-APC-H7 (clone MϕP9, BD Biosciences), Anti-CD16-PE (clone B73.1, BD Biosciences), Anti-HLA-DR-FITC (clone L243, BD Biosciences).

Dendritic Cell (DC) Panel: Anti-CD14-FITC (clone MϕP9, BD Biosciences), Anti-CD56-PE (clone NCAM16.2, BD Biosciences), Anti-CD11c-Percp-cy5.5 (clone B-1y6, BD Biosciences), Anti-CD19-PE (clone HIB19, BD Biosciences), Anti-CD123-APC (clone 9F5, BD Biosciences), Anti-CD3-APC-H7 (clone SK7, BD Biosciences), Anti-HLA-DR-V450 (clone L243, BD Biosciences).

### T cell proliferation assay

Fresh PBMCs were suspended in pre-warmed PBS at 3 × 10^6^ cells/mL and labeled with Cell Proliferation Dyes (CPD) at 10 μM in a 1:1 cell-to-dye ratio at 37 °C for 10 minutes. Labeled cells were washed with fresh medium containing 10% fetal bovine serum (FBS) and penicillin/streptomycin (100 U/mL), counted, seeded in 96-well plates (3 × 10^5^ cells/well), and stimulated with Dynabeads^®^ Human T-Activator CD3/CD28-coated beads (Invitrogen Dynal, Oslo, Norway). Unstimulated cells (media only) and unstained cells served as controls. Cell cultures were incubated at 37 °C in 5% CO_2_ for 5 days. After incubation, cells were harvested, washed, and analyzed by flow cytometry. Proliferation was assessed by dilution of the CPD dye. Data were analyzed using FlowJo software.

### Statistical analysis

Data are presented as mean ± standard deviation (SD) or mean ± SEM as indicated. Comparisons between patients and controls were performed using unpaired t-test with Welch’s correction for unequal variances. Paired t-test was used for before-and-after comparisons within the same patient. For flow cytometry data, statistical significance was determined using Mann-Whitney U test for non-normally distributed data. All statistical analyses were performed using GraphPad Prism version 9.0 (GraphPad Software). A p-value <0.05 was considered statistically significant.

## Results

### Clinical presentation

**Figure 1:**
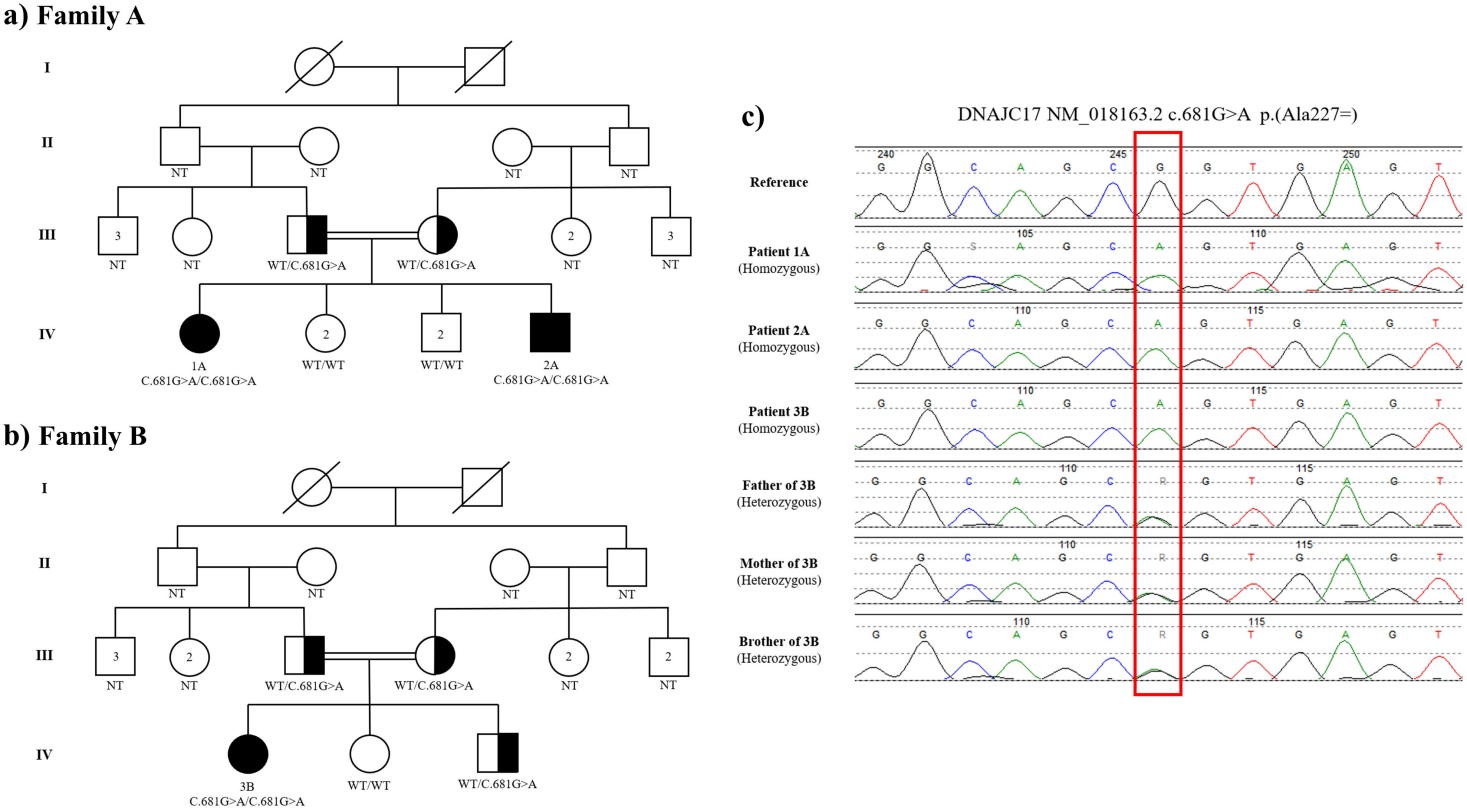
Pedigrees of the reported patients harboring the *DNAJC17* c.681G>A homozygous variant. **(a)** Family of siblings 1A and 2A. **(b)** Family of patient 3B. **(c)** Sanger sequencing chromatograms showing the variant in the affected individuals and segregation among family members of patient 3B.

Patients 1A and 2A are siblings from a consanguineous family (**Figure 1a**), a 35-year-old female (1A) and her 26-year-old brother (2A), who presented with a consistent phenotype characterized by early-onset retinitis pigmentosa diagnosed in infancy, recurrent infections, and persistent lymphadenopathy. Initial immunologic evaluation at age of 2 years revealed hypogammaglobulinemia (IgG <200 mg/dL; reference 400–1000 mg/dL for age), prompting initiation of regular intravenous immunoglobulin (IVIg) replacement therapy (400–600 mg/kg every 4 weeks). Patient 1A’s course was complicated by recurrent sinopulmonary infections requiring multiple hospitalizations during childhood. She developed progressive visual impairment beginning in the first year of life, with complete blindness by 12 years of age. Ophthalmologic examination revealed characteristic retinitis pigmentosa with bone-spicule pigmentation, arteriolar attenuation, and optic disc pallor. Beginning at age 10 years, she experienced recurrent febrile episodes (temperature >38.5 °C) lasting 5–10 days, occurring every 4–8 weeks, accompanied by tender cervical and axillary lymphadenopathy. Extensive infectious workup including blood cultures, viral PCR panels, tuberculosis testing, and fungal serologies were consistently negative. Patient 2A presented similarly with early-onset retinitis pigmentosa and hypogammaglobulinemia. At age 8 years, he developed severe meningoencephalitis of unclear etiology complicated by ischemic stroke affecting the left middle cerebral artery territory, resulting in right-sided hemiparesis, severe lower-limb spasticity, contractures, and wheelchair dependence. Like his sister, he developed recurrent unexplained febrile episodes associated with generalized lymphadenopathy. Genetic testing identified a homozygous synonymous variant in *DNAJC17* (NM_018163.2:c.681G>A; p.Ala227=) located at the last nucleotide of exon 9, adjacent to the highly conserved donor splice site. Sanger sequencing confirmed the presence of the variant in both patients (**Figure 1c**). Although this substitution does not alter the encoded amino acid, its position strongly suggested an effect on pre-mRNA splicing. RT-PCR analysis of RNA extracted from patient-derived lymphoblastoid cells confirmed skipping of exon 9, validating the predicted splice-site defect (8). The siblings were referred to our clinic for evaluation of the recurrent febrile episodes and lymphadenitis. On examination, both exhibited generalized lymphadenopathy, splenomegaly, and complete blindness due to progressive retinal degeneration. Laboratory investigations demonstrated persistently elevated inflammatory markers including CRP levels of 45–120 mg/L (reference <8 mg/L), and ESR of 80–110 mm/hr (reference <20 mm/hr). Formal vaccine-specific antibody testing was not performed as both patients were already established on immunoglobulin replacement therapy at the time of our comprehensive immunological evaluation.

The third patient (3B) is a 19-year-old girl from another consanguineous family (**Figure 1b**) who presented with a remarkably similar phenotype. Her symptoms were first noted at 19 days of life when she developed fever (39.2 °C) associated with cervical lymphadenitis and panniculitis-like skin lesions on the trunk and extremities. Skin biopsy showed lobular panniculitis with lymphocytic infiltration. Subsequently, she developed recurrent sinopulmonary infections, sterile osteomyelitis, and multiple episodes of sterile superficial and deep soft tissue abscesses requiring incision and drainage. Early-onset retinitis pigmentosa was diagnosed at 18 months of age during evaluation for poor visual tracking. Progressive visual deterioration led to complete vision loss by age 10 years. Beginning at age 3 years, the patient developed recurrent febrile episodes (temperature 38.5-40 °C) lasting 7–14 days, occurring every 3–6 weeks, accompanied by cervical and axillary lymphadenopathy, arthralgia, and profound fatigue. These attacks were often severe enough to require multiple hospitalizations. Extensive infectious evaluation including blood cultures, viral studies, fungal testing, and tuberculosis workup was consistently unrevealing.

Similar to patients 1A and 2A, initial immunologic assessment demonstrated hypogammaglobulinemia, persistent elevation of inflammatory markers during symptomatic periods (CRP 80–154 mg/L, ESR 90–120 mm/hr), and elevated circulating cytokines such as TNF-α (14 pg/ml; reference: < 0.1 pg/ml) and IL-6 (48 pg/ml; reference: < 7 pg/ml). She was found to have hypogammaglobulinemia with low baseline titers to Hepatitis B surface antigen antibody (HBsAb) and tetanus toxoid IgG antibody. Following administration of a DTP (diphtheria-tetanus-pertussis) booster vaccine, antibody titer transiently increased to normal/protective level however, this titer subsequently declined over time back to sub-protective levels.

On examination, she was found to have prominent growth failure with weight and height falling below the 3^rd^ percentile, tender bilateral cervical lymphadenopathy, splenomegaly, multiple hypopigmented scars secondary to prior panniculitis and abscess drainage procedures, and complete blindness with characteristic retinitis pigmentosa findings. WGS demonstrated a homozygous synonymous variant in *DNAJC17* (c.681G>A) identical to the mutation in Family A. Segregation analysis using bi-directional Sanger sequencing confirmed the presence of the variant in the index case while parents and her younger brother (age 10 years, clinically unaffected) were heterozygous carriers, and her older sister (age 18 years, clinically unaffected) carried the wild-type genotype (**Figure 1c**).

Comprehensive laboratory investigations, including complete blood count (CBC), renal, liver, and thyroid profiles for the three reported patients, are summarized in **Supplementary Tables 1** and **S2**.

### Molecular characterization of *DNAJC17* variant

**Figure 2:**
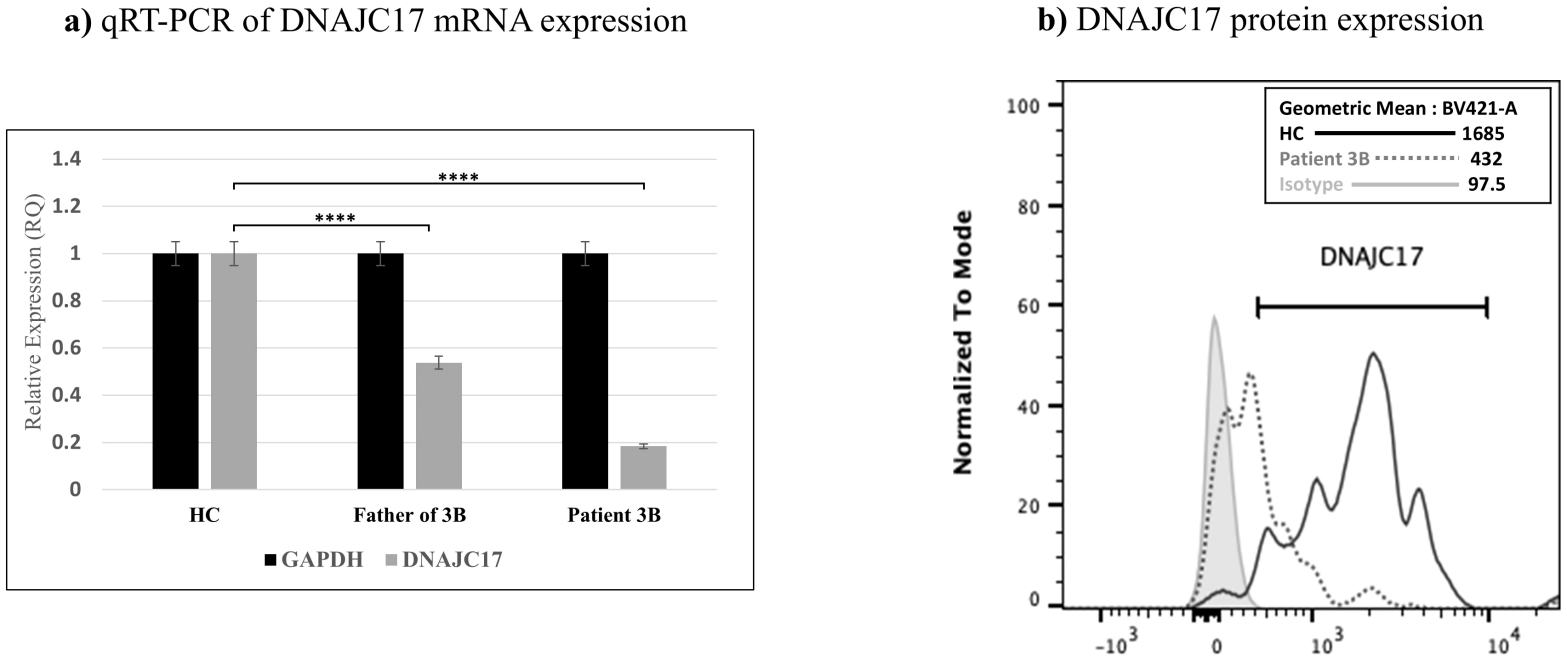
DNAJC17 expression analysis. **(a)** Quantitative real-time PCR (qRT-PCR) analysis of DNAJC17 mRNA expression in a healthy control (HC), Patient 3B, and her father. Relative DNAJC17 transcript levels were normalized to an internal housekeeping gene (GAPDH) and are presented as relative expression values. *p* < 0.0001 (****). **(b)** Flow cytometric analysis of DNAJC17 expression in viable peripheral blood mononuclear cells from a healthy control (HC; black solid line) and Patient 3B with DNAJC17 deficiency (dashed line), shown as mean fluorescence intensity (MFI). The isotype control is presented as a gray shaded area.

WGS was reviewed for the three patients to exclude other monogenic variants associated with IEI that could explain the phenotype. No additional relevant variants were identified apart from the homozygous *DNAJC17* (c.681G>A) variant. To investigate the molecular consequences of the c.681G>A variant at the mRNA level, we performed quantitative RT-PCR on PBMCs from patient 3B, her father and an age-matched healthy control. DNAJC17 mRNA levels were reduced in the heterozygous father compared to the healthy control and were significantly decreased in the homozygous patient (**Figure 2a**).

Flow cytometric analysis of intracellular DNAJC17 expression in viable PBMCs demonstrated markedly reduced protein expression in the patient 3B compared with the healthy control (**Figure 2b**). The control sample showed a clear rightward shift in fluorescence intensity (geometric mean fluorescence intensity MFI: 1685), whereas the patient sample exhibited a left-shifted distribution with substantially lower signal intensity (MFI: 432), approaching the isotype control (MFI: 97.5). These findings indicate significantly decreased DNAJC17 protein expression in the patient, supporting the functional impact of the identified homozygous *DNAJC17* variant.

According to ACMG/AMP criteria, this variant meets the criteria of likely pathogenic in a gene for which loss of function is an established disease mechanism, as evidenced by: (1) This variant affect the last nucleotide of exon 9, a critical position for correct RNA splicing which is confirmed by RT-PCR showing skipping of exon 9; (2) the critical location of the deleted region within RNA Recognition Motif (RRM), the C-terminal functional domain responsible for RNA substrate binding; (3) the variant was detected in homozygous state in three affected individuals from two unrelated consanguineous families with identical phenotypes, and all heterozygous carriers (parents and unaffected siblings) are clinically normal, consistent with recessive inheritance; (4) the combination of retinitis pigmentosa, autoinflammation, and hypogammaglobulinemia represent a phenotypically rare and unique presentation to DNAJC17-related disease; (5) finally, the variant is absent in homozygous state in Genome Aggregation Database (gnomAD) v3.1.2 (8 heterozygous individuals out of 76,156 total, allele frequency = 0.0000526, zero homozygotes).

### Comprehensive immunological characterization

**Figure 3:**
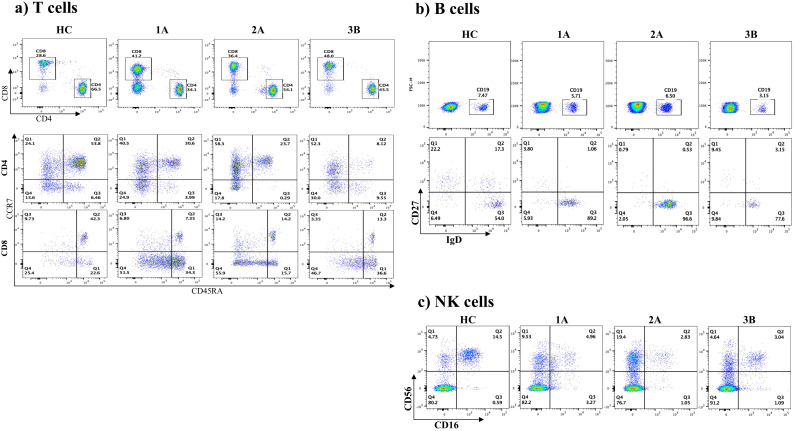
Flow cytometric analysis of lymphocyte subsets. **(a)** CCR7 and CD45RA expression on CD4^+^ and CD8^+^ T cells, **(b)** CD27 and IgD expression on CD19^+^ B cells, and **(c)** CD56 and CD16 expression on total lymphocytes identifying natural killer (NK) cell populations. Data are shown for a healthy control (HC) and patients 1A, 2A, and 3B.

**Figure 4:**
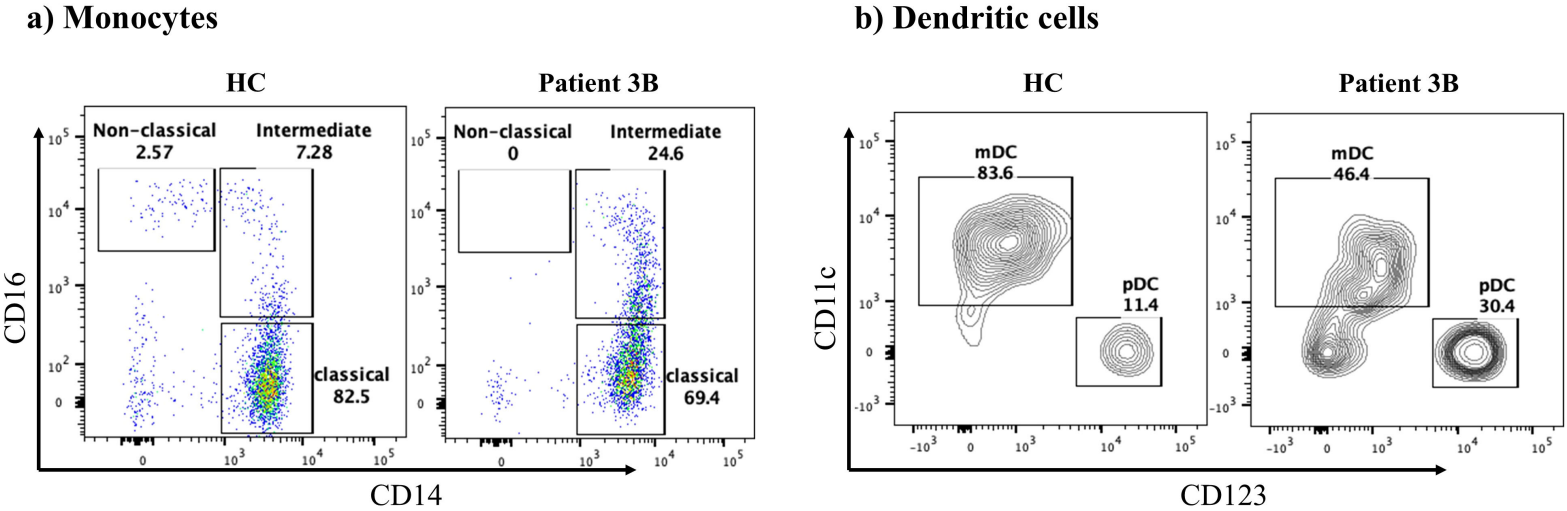
Flow cytometric analysis of immune cell subsets. **(a)** Monocyte subsets were identified based on CD14 and CD16 expression levels and classified as classical, intermediate, and non-classical monocytes within CD3^-^CD19^-^ (dump gate) and HLA-DR^+^ cells. **(b)** Dendritic cell (DC) subsets were identified based on CD11c and CD123 expression levels and classified as CD11c^+^CD123^-^ myeloid dendritic cells (mDCs) and CD11c^-^CD123^+^ plasmacytoid dendritic cells (pDCs) within CD3^-^CD19^-^CD56^-^CD14^-^ (dump gate) and HLA-DR^+^ cells. Data are shown for a healthy control (HC) and patient 3B.

Flow cytometric analysis of peripheral blood lymphocytes from all three patients revealed distinct abnormalities in T, B, and NK-cell subsets (**Figure 3**). T-cell analysis showed normal CD3^+^ T-cell counts; however, there was a skewing of the CD4:CD8 ratio with relative CD8^+^ T-cell predominance in patients 1A and 3B. There was a significant decrease in naïve CD4^+^ and CD8^+^ T cells (CCR7^+^CD45RA^+^), with a concomitant expansion of central (CCR7^+^CD45RA^-^) and effector memory subsets (CCR7^-^CD45RA^-^) (**Figure 3a**), indicating chronic immune activation and impaired T-cell renewal. B-cell profiling demonstrated an increased frequency of naïve (IgD^+^CD27^-^) B cells accompanied by a marked reduction in both unswitched (IgD^+^CD27^+^) and switched memory (IgD^-^CD27^+^) B cells (**Figure 3b**). In addition, all patients exhibited a pronounced reduction in CD56^+^CD16^+^ cytotoxic NK cells (**Figure 3c**). Given the prominent autoinflammatory features, we performed comprehensive analysis of innate immune cell populations including monocytes and dendritic cells (**Figure 4**). In compare to a healthy control, there is a significant reduction of non-classical monocytes (CD14^-^CD16^+^), with a clear expansion of intermediate monocytes (CD14^+^CD16^+^), a subset associated with inflammatory conditions and elevated TNF-α production. Monocytes also displayed elevated expression of the activation marker HLA-DR indicating constitutive monocyte activation. Additionally, dendritic cell subsets analysis demonstrated a marked reduction in myeloid dendritic cells (mDCs), accompanied by a relative expansion of plasmacytoid dendritic cells (pDCs), which may suggest enhanced interferon-driven immune activation.

**Figure 5:**
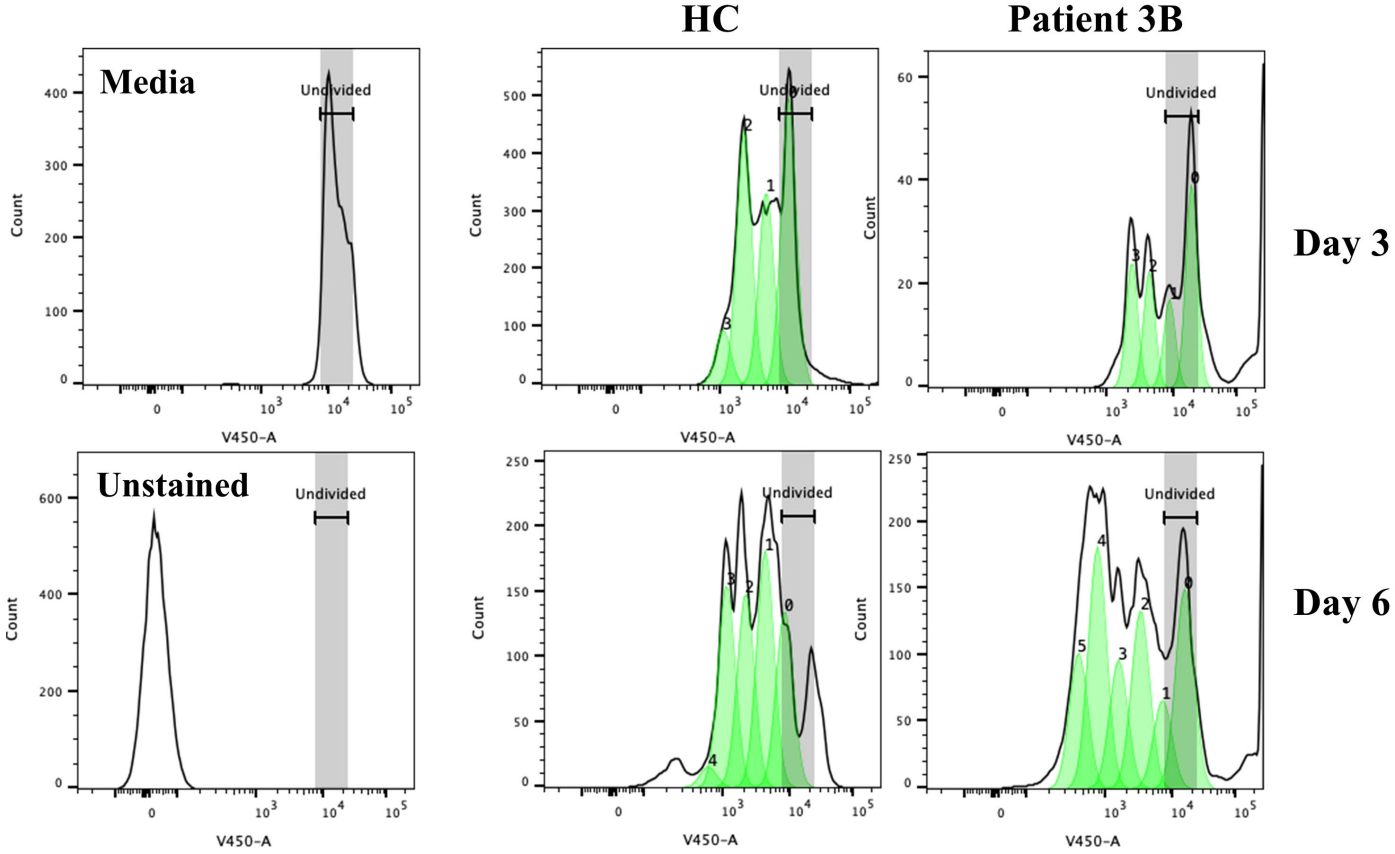
Proliferation analysis of peripheral blood mononuclear cells (PBMCs) from a healthy control (HC) and patient 3B. Cells were stained with V450 dye and stimulated with CD3/CD28 beads for 3 or 6 days. Unstained PBMCs (Unstained) and stained but unstimulated PBMCs (Media) were included as controls.

To further define the immunological phenotype associated with DNAJC17 deficiency, we assessed lymphocyte proliferative capacity in patient 3B. PBMCs stimulated with CD3/CD28-coated beads displayed normal proliferative responses at both day 3 and day 6 post-stimulation (**Figure 5**), indicating preserved T-cell activation and proliferation despite the skewed memory and naïve cell distribution. This finding suggests that the immunodeficiency in patients with DNAJC17 deficiency primarily reflects impaired lymphocyte development, differentiation, or survival rather than intrinsic defects in T-cell receptor signaling or proliferative machinery.

**Figure 6:**
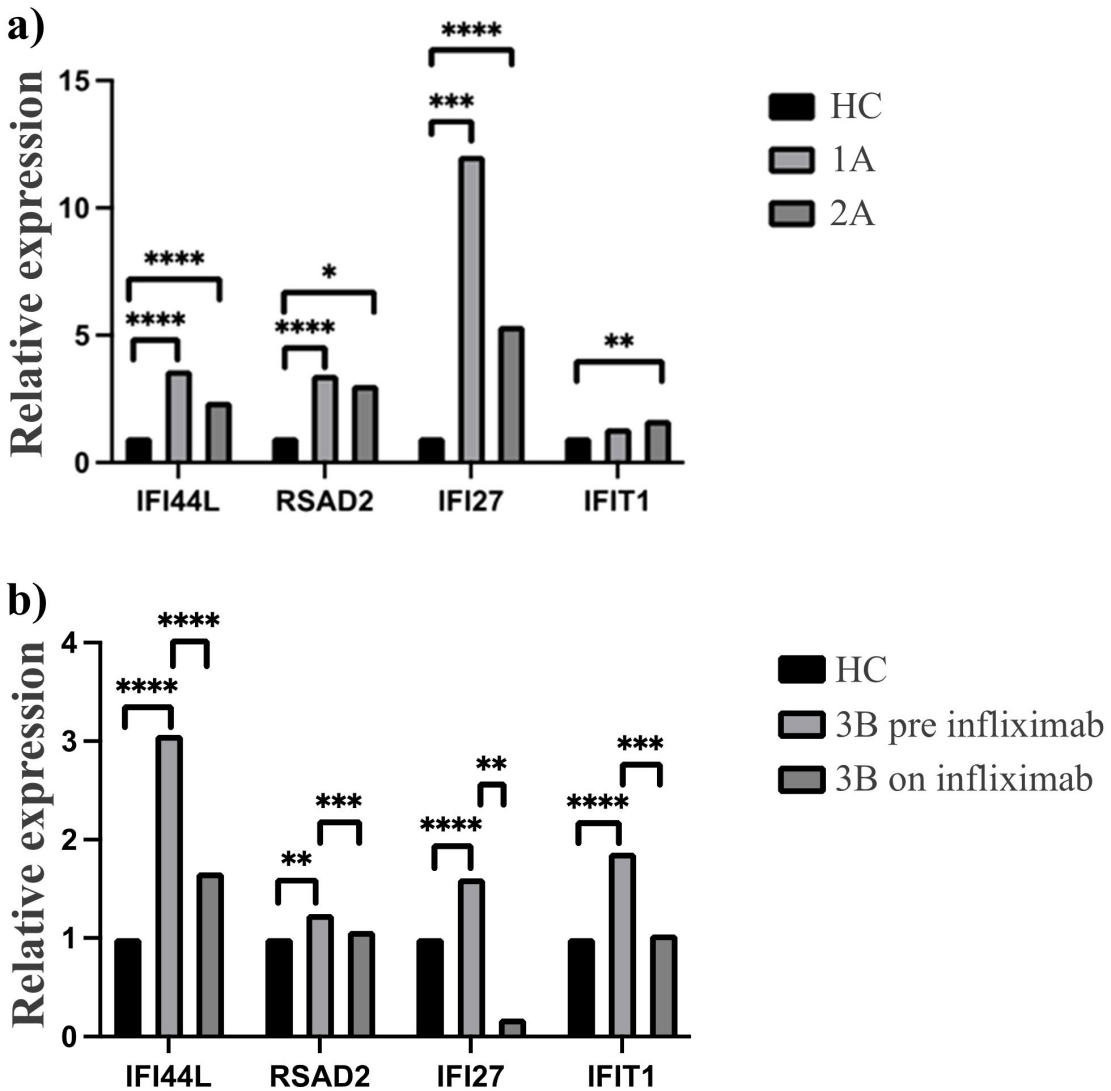
Expression of interferon-stimulated genes (ISGs). **(a)** ISGs (IFI44L, RSAD2, IFI27, and IFIT1) expression in a healthy control (HC) and patients 1A and 2A. **(b)** Relative mRNA expression of ISGs in a healthy control (HC) and patient 3B pre- and during infliximab treatment. Increased ISG expression was observed in patients compared with healthy controls. *p* < 0.05 (*), p < 0.01 (**), p < 0.001 (***), *p* < 0.0001 (****).

Given the patient’s panniculitis-like skin lesions, a feature often observed in interferonopathies, a quantitative assessment of interferon-stimulated gene (ISG) expression was performed, revealing a modest but consistent upregulation of multiple ISGs (IFI44L, RSAD2, IFI27, IFIT1) compared to healthy controls (p <0.05 for all genes tested). However, the magnitude of ISG upregulation was substantially lower than that observed in classic type I interferonopathies, suggesting that interferon dysregulation is a secondary feature rather than the primary driver of disease pathogenesis (**Figure 6**).

### Therapeutic interventions and outcomes

Initial therapeutic approaches included prolonged courses of antibiotics for presumed bacterial infections, and monthly IVIg infusions without noticeable improvement. Corticosteroids provided transient symptomatic relief in all patients but were associated with significant side effects and loss of efficacy with dose reduction. Given the elevated inflammatory markers and fever, IL-1 blockade was attempted, resulting in minimal clinical improvement and persistent elevation of inflammatory markers. Patient 3B was started on colchicine (maximum total daily dose: 2 mg/day) for six months and a course of JAK inhibitor (ruxolitinib; maximum total daily dose: 30 mg/day) with no significant clinical benefit.

Based on the markedly elevated TNF-α levels, inflammatory phenotype, and expansion of intermediate monocytes, TNF inhibitor therapy was proposed as a targeted, mechanism-based intervention. After extensive discussion of risks and benefits, including the concern about TNF inhibition in the context of underlying immunodeficiency, the family of patient 3B consented to a trial of infliximab. Patients 1A and 2A declined TNF inhibitor therapy due to concerns about immunosuppression and previous adverse experiences with immunomodulatory medications.

Infliximab (10 mg/kg every 4 weeks) was initiated in patient 3B, resulting in a rapid and dramatic clinical response, including complete resolution of fever and reduction in lymphadenopathy. Laboratory evaluation demonstrated normalization of CRP (from 154 mg/L to 6 mg/L; reference: <8 mg/L) and ESR (from 120 mm/hr to 3 mm/hr; reference: <20 mm/hr). Serum TNF-α, IL-6 levels and ISG expression decreased to near-normal ranges (**Supplementary Table 1**, **Figure 6****).**

The patient was closely monitored with routine laboratory follow-up, including complete blood counts and inflammatory markers (CRP and ESR), performed monthly during the initial treatment period and subsequently every three months thereafter. These evaluations remained within normal ranges throughout follow-up. During this time, the patient remained clinically stable with sustained remission and no recurrence of inflammatory episodes. To date, the patient has maintained remission for over 3 years while receiving infliximab therapy, without significant adverse events.

## Discussion

This study establishes DNAJC17 deficiency as a novel monogenic IEI characterized by combined immunodeficiency, retinopathy, and TNF-driven autoinflammation responsive to targeted therapy. Our comprehensive immunological characterization reveals a complex phenotype involving multiple immune compartments and provides mechanistic insights with direct therapeutic implications.

DNAJC17 belongs to the DNAJ/HSP40 family of co-chaperones that play critical roles in protein folding, quality control, and cellular stress responses (3). It encodes a J-domain-containing co-chaperone that functions as an essential component of the mitochondrial protein import machinery. Specifically, DNAJC17 is part of the presequence translocase-associated motor (PAM) complex, which facilitates the import and folding of nuclear-encoded proteins into the mitochondrial matrix (3, 4). The J-domain of DNAJC17 stimulates the ATPase activity of mitochondrial HSP70 (HSPA9/mortalin), driving the power stroke that pulls preproteins across the inner mitochondrial membrane. This process is fundamental to mitochondrial biogenesis and the maintenance of mitochondrial proteostasis (14).

The critical importance of DNAJC17 is underscored by the embryonic lethality observed in complete *Dnajc17* knockout mice, which die before blastocyst stage (4, 5). The viability of our patients carrying the hypomorphic c.681G>A variant suggests that the exon 9-skipped transcript retains partial function, sufficient for embryonic development but inadequate for normal immune system function. Our findings of reduced but not absent DNAJC17 mRNA and protein expression support this hypothesis of hypomorphic rather than null function. These findings align with growing evidence that synonymous variants can be pathogenic by altering splicing, mRNA stability, or translation efficiency (12, 15, 16). Collectively, such mechanisms emphasize the importance of comprehensive variant interpretation that extends beyond simple amino acid substitution predictions.

The connection between DNAJC17 deficiency and immunodeficiency can be understood through the critical dependence of lymphocytes on mitochondrial function. T cell differentiation and activation require rapid metabolic reprogramming with dramatic increases in both glycolysis and oxidative phosphorylation (17, 18). Impaired DNAJC17 function would compromise this process, potentially explaining the T cell subset abnormalities we observe, particularly the depletion of naïve T cells and expansion of memory populations indicating chronic activation and impaired T cell homeostasis. The normal T cell proliferative response observed in patient 3B despite the immunological abnormalities suggests that the immunodeficiency primarily reflects impaired lymphocyte development, differentiation, or survival rather than intrinsic defects in T cell receptor signaling or proliferative capacity. This finding is consistent with a model in which DNAJC17 deficiency causes chronic cellular stress that preferentially affects cells with high metabolic demands during specific developmental or activation stages. Similarly, B cell differentiation into antibody-secreting plasma cells is one of the most energy-intensive cellular processes in the immune system, with immunoglobulin production consuming vast amounts of ATP (19). The hypogammaglobulinemia and marked reduction in switched memory B cells in our patients are consistent with an inability to meet these metabolic demands during B cell activation and differentiation. The requirement for IVIg replacement therapy underscores the clinical significance of this antibody deficiency. Additionally, NK cell cytotoxicity depends heavily on mitochondrial function for both energy supply and regulation of apoptotic pathways (20). The pronounced reduction in NK cells, particularly the cytotoxic CD56^+^CD16^+^ subset, in all three patients suggests impaired NK cell development or survival in the context of mitochondrial dysfunction.

The phenotype of DNAJC17 deficiency is parallel to other mitochondrial disorders (e.g. TRNT1 deficiency) with combined immunodeficiency and autoinflammation, validating the concept that mitochondrial homeostasis is essential for balanced immunity (21). These disorders share the common theme of mitochondrial dysfunction driving both impaired lymphocyte function and chronic inflammation, supporting a unified pathophysiological framework. The autoinflammatory phenotype with markedly elevated TNF-α and IL-6, together with the marked clinical response to TNF blockade, provides compelling evidence that TNF-α plays a central role in driving the inflammatory phenotype. Our demonstration that DNAJC17 deficiency is sufficient to induce TNF-α production in monocytic cells provides strong mechanistic support for a direct causal relationship between DNAJC17 deficiency and TNF-α-driven inflammation. The magnitude of TNF-α induction is supporting the biological relevance of this finding. The expansion of intermediate monocytes (CD14^+^CD16^+^), a subset known to be a major source of TNF-α in inflammatory conditions (22), further supports this mechanism. In addition, the observed reduction in mDCs with relative enrichment of pDCs suggests disrupted dendritic cell homeostasis in DNAJC17 deficiency, potentially favoring aberrant type I interferon signaling and impaired antigen presentation. The modest upregulation of ISGs further supports activation of innate immune sensing pathways, possibly as a consequence of cellular stress or defective protein homeostasis. Notably, the absence of a robust type I interferon signature distinguishes this condition from classical primary interferonopathies.

We acknowledge several limitations in our study. The small number of patients reflects the rarity of this condition but limits statistical power for certain analyses. Long-term follow-up data on additional patients treated with TNF inhibitors will be important to establish the generalizability of this therapeutic approach. It is important to identify biomarkers for disease monitoring and treatment response and to have long-term assessment of TNF inhibitor efficacy and safety in this patient population.

In conclusion, we identify DNAJC17 deficiency as a novel inborn error of immunity characterized by retinopathy, combined immunodeficiency, and TNF-driven autoinflammation. The pathogenic c.681G>A variant leads to aberrant splicing with reduced DNAJC17 mRNA expression and consequent decrease in intracellular protein levels, confirming a hypomorphic loss-of-function mechanism. This molecular defect underlies profound immune dysregulation affecting adaptive and innate compartments. Importantly, targeted TNF blockade resulted in sustained clinical and biochemical remission, establishing a direct mechanistic link between DNAJC17 deficiency and inflammatory pathology and highlighting a precision-medicine approach for affected patients.

## Data Availability

The original contributions presented in the study are included in the article/**Supplementary Material**. Further inquiries can be directed to the corresponding author.
