# Supplementary material for: DNAJC17 deficiency: A novel inborn error of immunity with TNF-driven autoinflammation
Source: Front Immunol. 2026 Jul 9;17:1751990. doi: 10.3389/fimmu.2026.1751990 (PMC13392675; doi:10.3389/fimmu.2026.1751990)
Supplement: Supplementary file 1 [file DataSheet1.pdf]

Supplementary Information

Table S1. Summary of clinical manifestations and laboratory findings in the reported patients (1A, 2A, and 3B).

Patient	1A	2A	3B		Normal Range
			Pre-TNFi	On-TNFi	
**Clinical Features**					
Sex	Female	Male	Female		
Disease onset (months)	4	6	< 1		
Current age (years)	35	26	19		
Retinitis pigmentosa	+	+	+	+	
Recurrent fever	+	+	+	-	
Sinopulmonary infections	+	+	+	+	
Recurrent bacteremia	+	-	-	-	
Meningoencephalitis	-	+	-	-	
Osteomyelitis	-	-	+	-	
Soft tissue infections	+	+	+	-	
Lymphadenitis	+	+	+	-	
Growth failure	+	+	+	+	
High IMs	+	+	+	-	
Hypogammaglobulinemia	+	+	+	+	
Current treatment	IVIg	IVIg	Infliximab, Methotrexate, IVIg		
Outcome	Recurrent fever with high IMs, requiring intermittent courses of steroid		Complete remission		
**Peripheral blood analysis**					
White blood cells (cells/L)	5.41	5.46	7.53	6.52	4 – 11 (10^9 cells/L)
Hemoglobin (gm/L)	112	105	82	121	110 – 145 gm/L
Platelets (cells/L)	313	235	491	345	150 – 450 (10^9 cells/L)
Lymphocytes (cells/mL)	0.96	1.31	3.13	3.5	1.4 – 8.4 x10^9/L
Neutrophils (cells/mL)	3.89	3.27	2.24	1.59	0.8 – 5.4 x10^9/L
Monocytes (cells/mL)	0.41	0.73	0.91	0.76	0.1 – 1.1 x10^9/L
**Inflammatory markers**					
ESR (mm/hr)	102	104	120	3	0 – 20 mm/hr
C-reactive protein (mg/L)	86.8	82.5	154	6	< 8 mg/L
**Liver, renal and thyroid profile**					
Aspartate transferase (U/L)	14	21	13	13	5 – 34 U/L
Alanine aminotransferase (U/L)	34	43	8	11	5 – 55 U/L
GGT (U/L)	21	32	19.9	30.6	9 – 36 U/L
Albumin (g/L)	40	39	44	45	38 – 54 g/L
Creatinine (umol/L)	55	45	47	36	27 – 62 umol/L
Blood urea nitrogen (mmol/L)	5.3	4.8	3.1	2.8	2.5 – 6 mmol/L

TSH (mIU/L)	2.1	2.8	1.9	NT	0.5 – 5 mIU/L
Free T4 (ng/dL)	1.2	1.4	1.3	NT	0.8 – 1.8 ng/dL
Free T3 (pg/mL)	3.1	2.9	3.4	NT	2.3 – 4.2 pg/mL
**Others**					
Antinuclear antibody (unit)	3.2	1.3	1.86	NT	< 20 unit is negative
TNF alpha (pg/mL)	NT	NT	14	< 0.1	Up to 0.1 pg/ml
Interleukin 6 (pg/mL)	NT	NT	48	< 1	~ 7 pg/mL
Anti-cardiolipin antibody, IgG	NT	NT	5.5	-	< 15 GPL unit
Anti-cardiolipin antibody, IgM	NT	NT	10.1	-	< 12.5 MPL unit
B2 glycoprotein I IgG	NT	NT	0.5	-	< 20 SGU
B2 glycoprotein I IgM	NT	NT	0.51	-	< 20 SMU
Lupus anticoagulant	NT	NT	Negative	-	Negative
Complement C3 (g/L)	NT	NT	1.98	1.1	0.79 – 1.52 g/L
Complement C4 (g/L)	NT	NT	0.42	0.24	0.16 – 0.38 g/L
Total Complement CH50 (U/mL)	NT	NT	49	-	31 – 60 U/mL

IMs; Inflammatory markers, ESR; erythrocyte sedimentation rate, GGT; Gamma-glutamyl transferase, TSH; Thyroid Stimulating Hormone, TNF; Tumor Necrosis Factor, NT; Not tested.

Table S2. Lymphocyte subsets and immunoglobulin levels for the reported patients (1A, 2A, and 3B).

Patient	1A # (%)	2A # (%)	3B # (%)	Normal Range
**Lymphocyte subsets**				
WBC Count (cells/mcL)	5410	5460	7530	4000 – 11000
$CD3^+$ (cells/mcL)	851 (88)	1152 (88)	2463 (78.7)	1500 – 5700
$CD3^+CD4^+$ (cells/mcL)	441 (46)	534 (40.8)	1255 (40.1)	1100 – 4100
$CD3^+CD8^+$ (cells/mcL)	271 (28.3)	508 (38.8)	1208 (38.6)	600 – 2400
$CD19^+$ (cells/mcL)	63 (6.6)	85 (6.5)	175 (5.6)	200 – 600
$CD3^-CD16^+CD56^+$ (cells/mcL)	50 (5.3)	77 (5.9)	62 (2)	200 – 500
$CD3^+$TCR gamma/delta	171 (17.9)	250 (19.1)	560 (17.9)	–
$CD3^+CD45RA^+$	353 (36.8)	442 (33.8)	2022 (64.6)	–
$CD3^+CD45RO^+$	407 (42.4)	590 (45.1)	1333 (42.6)	–
$CD3^+HLADR^+$	(18.3)	(22.5)	(3.8)	–
$CD19^+HLADR^+$	(6.6)	(6.5)	(5.6)	–
**Immunoglobulin levels**				
Immunoglobulin A (g/L)	1.9	0.79	0.29	0.82 – 4.53 g/L
Immunoglobulin G (g/L)	14.6*	12.8*	5.88	7.51 - 15.6 g/L
Immunoglobulin M (g/L)	0.27	0.12	0.31	0.5 – 3.04 g/L
HBsAb	NT	NT	0	≥ 10 mIU/mL (protective)
Tetanus Toxoid IgG	NT	NT	0.02	≥ 0.1 IU/mL (protective)
Oxidative Burst Test	NT	NT	Normal	–

NT; Not tested, HBsAb; Hepatitis B surface antigen antibody, #; cell count number, %; cell percentage. *On immunoglobulin replacement.
